# Generation and Characterization of a Human/Mouse Chimeric GD_2_-Mimicking Anti-Idiotype Antibody Ganglidiximab for Active Immunotherapy against Neuroblastoma

**DOI:** 10.1371/journal.pone.0150479

**Published:** 2016-03-11

**Authors:** Christin Eger, Nikolai Siebert, Diana Seidel, Maxi Zumpe, Madlen Jüttner, Sven Brandt, Hans-Peter Müller, Holger N. Lode

**Affiliations:** 1 Department of Pediatric Oncology and Hematology, University Medicine Greifswald, 17475, Greifswald, Germany; 2 ZIK HIKE, Center for Innovation Competence: Humoral Immune Reactions in Cardiovascular Diseases, University of Greifswald, Greifswald, Germany; Gustave Roussy, FRANCE

## Abstract

Vaccination with proteins mimicking GD_2_ that is highly expressed on neuroblastoma (NB) cells is a promising strategy in treatment of NB, a pediatric malignancy with poor prognosis. We previously showed efficacy of ganglidiomab *in vivo*, a murine anti-idiotype (anti-Id) IgG1. In order to tailor immune responses to variable regions, we generated a new human/mouse chimeric anti-Id antibody (Ab) ganglidiximab by replacing murine constant fragments with corresponding human IgG1 regions. DNA sequences encoding for variable regions of heavy (VH) and light chains (VL) were synthesized by RT-PCR from total RNA of ganglidiomab-producing hybridoma cells and further ligated into mammalian expression plasmids with coding sequences for constant regions of human IgG1 heavy and light chains, respectively. We established a stable production cell line using Chinese hamster ovarian (CHO) cells co-transfected with two expression plasmids driving the expression of either ganglidiximab heavy or light chain. After purification from supernatants, anti-idiotypic characteristics of ganglidiximab were demonstrated. Binding of ganglidiximab to anti-GD_2_ Abs of the 14.18 family as well as to NK-92tr cells expressing a GD_2_-specific chimeric antigen receptor (scFv(ch14.18)-zeta) was shown using standard ELISA and flow cytometry analysis, respectively. Ganglidiximab binding affinities to anti-GD_2_ Abs were further determined by surface plasmon resonance technique. Moreover, binding of anti-GD_2_ Abs to the nominal antigen GD_2_ as well as GD_2_-specific Ab-mediated cytotoxicity (ADCC, CDC) was competitively inhibited by ganglidiximab. Finally, ganglidiximab was successfully used as a protein vaccine *in vivo* to induce a GD_2_-specific humoral immune response. In summary, we report generation and characterization of a new human/mouse chimeric anti-Id Ab ganglidiximab for active immunotherapy against NB. This Ab may be useful to tailor immune responses to the paratope regions mimicking GD_2_ overexpressed in NB.

## Introduction

NB is the most common extracranial solid tumor in early childhood. About 50% of patients show a high-risk NB phenotype, characterized by wide spread dissemination and poor long-term survival despite intensive multimodal treatments [1–3]. A successful approach to improve survival in high-risk NB patients is immunotherapy targeting the tumor-associated antigen (TAA) GD_2_ due to its high expression on NB cells [4, 5] and poor physiological expression on healthy tissues [6]. Therefore, monoclonal Abs (mAbs) directed against GD_2_ have been developed and successfully applied over the past two decades in a number of clinical trials for NB [7, 8]. However, one obstacle associated with infusion of ch14.18/CHO is pain toxicity [9] which correlates with infusion rate [10]. Recently, a novel treatment method based on a long term infusion of ch14.18/CHO in combination with interleukin-2 was initiated and showed a reduced toxicity profile [10]. Since passive immunotherapy does not induce a long lasting immune response, there is a considerable interest in extending these studies into active immunotherapy. However, active vaccination with GD_2_ encounters several significant drawbacks including its weak immunogenicity and T cell independence due to its glycolipid structure [11, 12]. A promising alternative strategy exploits the immune network hypothesis of Jerne [13] to present GD_2_ as a protein epitope by an anti-Id Ab acting as a T cell-dependent surrogate with enhanced immunogenicity. Therefore, TAA-mimicking anti-Id Abs are used for protein vaccine development and successfully applied in a number of preclinical and clinical studies for a variety of solid tumors [14, 15]. For treatment of high-risk NB patients, an anti-Id Ab 1A7 bearing the internal image of GD_2_ was developed [16] and used as protein vaccine [1]. Importantly, patients vaccinated with 1A7 showed little side effects which underlines the suitability of such a vaccine for clinical application. To provide unrestricted access for clinical development in Europe, we recently generated and characterized a murine anti-Id Ab ganglidiomab which paratopes mimic GD_2_. Vaccination of mice resulted in an induction of a GD_2_-specific humoral immunity [17]. To further tailor this immune response induced by ganglidiomab to GD_2_-mimicking paratopes in prospective clinical trials, we generated a chimeric human/mouse anti-Id Ab ganglidiximab by replacing murine constant regions with corresponding fragments of human IgG1.

Here, we report the generation and characterization of this new chimeric human/mouse GD_2_-mimicking anti-Id Ab providing an important baseline for the development of protein vaccines with clinical potential for active immunotherapy against NB.

## Materials and Methods

### Cell culture

A GD_2_-positive murine NB cell line NXS2 [18] and CHO cell line [19] (ATCC, Wesel, Germany) were cultured in Dulbecco's modified Eagle's medium supplemented with stable glutamine, 4.5 g/l glucose (DMEM; PAN-Biotech, Aidenbach, Germany), 10% (v/v) fetal calf serum (FCS), 100 U/ml penicillin and 0.1 mg/ml streptomycin (1× P/S; PAA, Pasching, Austria). Hybridoma cells producing murine anti-Id Ab ganglidiomab [17] were cultured in serum-free DMEM with stable glutamine and 4.5 g/l glucose supplemented with 1× non-essential amino acids (PAA, Pasching, Austria) and 50 μM β-mercaptoethanol (Sigma Aldrich, Steinheim, Germany). The human NB cell line LA-N-1 [20] was cultured in RPMI (PAN-Biotech, Aidenbach, Germany) supplemented with 4.5 g/l glucose, 2 mM stable glutamine (PAA, Pasching, Austria), 10% (v/v) FCS and 1× P/S. The genetically engineered NK-92-scFv(ch14.18)-zeta cell line (NK-92tr), expressing a GD_2_-specific chimeric antigen receptor derived from ch14.18, was kindly provided by Prof. W. Wels (Georg-Speyer Haus, Frankfurt, Germany) and cultured as previously described [21].

### Mice

Analysis of GD_2_-specific humoral immune response upon vaccination with ganglidiximab was performed in female A/J mice (10 weeks of age; Charles River Laboratories, Sulzfeld, Germany). Mice were housed in standard animal laboratories (12 h light/dark cycle) with free access to water and standard laboratory chow *ad libitum*. Animal experiments were approved by the animal welfare committee (Landesamt für Landwirtschaft, Lebensmittelsicherheit und Fischerei Mecklenburg-Vorpommern, Thierfelderstraße 18, 18059 Rostock, LALLF M-V/TSD/7221.3-1-011/11) and approved and supervised by the commissioner for animal welfare at the University Medicine Greifswald representing the Institutional Animal Care and Use Committee (IACUC).

### Design and construction of ganglidiximab expression plasmids

#### RNA isolation and RT-PCR analysis

Total RNA was isolated from 5× 10^6^ hybridoma cells producing murine anti-Id Ab ganglidiomab using the RNeasy^®^ Mini Kit (QIAGEN GmbH, Hilden, Germany) and concentration was determined spectrophotometrically (BioPhotometer plus, Eppendorf, Hamburg, Germany). 1 μg of total RNA was used for cDNA synthesis w SuperScript^®^ II Reverse Transcriptase (Invitrogen GmbH, Darmstadt, Germany) according to the manufacturer’s guidelines. PCR amplification of ganglidomab VH and -VL was performed using gene-specific primers designed with online primer design tool Primer3 to allow amplification of a 440 bp PCR-product. For ganglidiomab VH amplification forward primer sequence 5`-GGG*gcggccgc*CATGGCTGTCTTGGGGCTGCTCTTCT-3’ including *Not*I-HF restriction site (italic, lowercase) and the reverse primer sequence 5’-CCC*ctcga*GACGGTGACTGAGGTTCCTTGA-3’ including *Xho*I restriction site (italic, lowercase) were used. VH was amplified by 30 cycles consisting of +94°C (15 s) for denaturation, +81°C (15 s) for primer-specific annealing and +72°C (30 s) for elongation. For ganglidiomab VL amplification, gene-specific primers were designed as described above. Following primer sequences were used for amplification of a 420 bp PCR-product: 5’-GGG*gcggccgc*CATGAAGTTGCCTGTTAGGCTGTTG -3’ (forward primer including *Not*I-HF restriction site; italic, lowercase) and 5’- CCC*cgtacg*AGCCCGTTTGATTTCCAGCTT -3’ (reverse primer including *Bsi*WI restriction site; italic, lowercase). VL was amplified by 30 cycles consisting of +94°C (15 s) for denaturation, +73°C (15 s) for primer-specific annealing, and +72°C (30 s) for elongation. Finally, PCR prodcucts were analyzed by agarose gel electrophoresis.

#### Isolation of murine ganglidiomab variable region from agarose gel

PCR products were isolated from agarose gels using a Nucleo Spin^®^ Extract II Kit (Macherey-Nagel, Düren, Germany) according to the manufacturer’s guidelines. Briefly, DNA fragments were excised from agarose gel and lysis buffer was added followed by incubation at +50°C until gel slices were completely dissolved. Then, the sample was loaded onto a Nucleo Spin^®^ column containing a silica membrane binding DNA (centrifugation: 11,000× g, 1 min, RT). After a wash step (centrifugation: 11,000× g, 1 min, RT), the silica membrane was dried (centrifugation: 11,000× g, 2 min, RT) and bound DNA was eluted in *Aqua destillata (A*. *dest)* (50 μl; centrifugation: 11,000× g, 1 min, RT). DNA concentration was determined spectrophotometrically as described above.

#### Cloning of murine ganglidiomab variable region into pCR^®^2.1-TOPO^®^ plasmid

After purification, ganglidiomab VH and -VL were cloned into pCR^®^2.1-TOPO^®^ plasmids (LifeTechnologies GmbH, Darmstadt, Germany) according to the manufacturer’s guidelines. In brief, the PCR-product (1 μg) was incubated with provided salt solution, *A*. *dest* and pCR^®^2.1-TOPO^®^ plasmid (10 ng) for 5 min at RT. Then, One Shot^®^ TOP 10 chemically competent *E*. *coli* cells were added and incubated for 20 min on ice. For transformation, *E*. *coli* were subjected to heat-shock for 30 s at +42°C and immediately placed back on ice. Subsequently, S.O.C. outgrowth medium was added and *E*. *coli* were shaken horizontally (400 rpm) for 1 h at +37°C. Then, transformed *E*. *coli* were incubated overnight at +37°C on ampicillin (50 μg/ml) containing Luria/Miller (LB) agar plates (Roth, Karlsruhe, Germany) coated with X-gal in dimethylformamide (DMF) (40 mg/ml) for blue/white screening. Finally, 15 positive clones (white colonies) were selected and cultured overnight in 5 ml LB medium (Roth, Karlsruhe, Germany) supplemented with 50 μg/ml ampicillin (+37°C, shaking at 100 rpm).

#### Purification of pCR^®^2.1-TOPO^®^ plasmid containing murine ganglidiomab variable region

Plasmids of successfully transformed clones were isolated from *E*. *coli* cultures using NucleoSpin^®^ Plasmid Kit (Macherey-Nagel, Düren, Germany) according to the manufacturer's guidelines. Briefly, pelleted *E*. *coli* (centrifugation: 11,000× g, 30 s, RT) were resuspended in provided buffer and lysed for 5 min at RT followed by a neutralization step to ensure appropriate DNA binding conditions. After centrifugation (11,000× g, 5 min, RT), the supernatant was loaded onto a Nucleo Spin^®^ plasmid column containing a silica membrane binding plasmid DNA (centrifugation: 11,000× g, 1 min, RT). After two wash steps (centrifugation: 11,000× g, 1 min, RT), the silica membrane was dried (centrifugation: 11,000× g, 2 min, RT) and bound DNA was eluted in *A*. *dest* (centrifugation: 11,000× g, 1 min, RT). Finally, purified plasmids containing DNA fragments of expected molecular size were sequenced (LGC Genomics, Berlin, Germany).

#### Restriction analysis of pCR^®^2.1-TOPO^®^ plasmids containing murine ganglidiomab variable regions

For further cloning, ganglidiomab VH and -VL were excised from purified pCR^®^2.1-TOPO^®^ plasmids using respective restriction enzymes. For ganglidiomab VH, plasmid DNA (4 μg) was incubated with *Not*I-HF (1 unit; New England Biolabs GmbH, Frankfurt/Main, Germany) and *Xho*I-HF (1 unit; New England Biolabs GmbH, Frankfurt/Main, Germany) in CutSmart^®^ Buffer (1×) for 1 h at +37°C followed by enzyme heat inactivation (20 min, +65°C). Resulting plasmid DNA products were analyzed by agarose gel electrophoresis and ganglidiomab VH DNA fragment (427 bp) was purified from agarose gel and spectrophotometrically quantified as described above. For ganglidiomab VL, double restriction digest using both *Not*I-HF and *Bsi*WI was not recommended due to different optimum incubation temperatures. Therefore, plasmid DNA (8 μg) was first linearized using *Not*I-HF (2 units) followed by purification from agarose gel as described above. Then, linearized plasmid DNA (4 μg) was incubated with *Bsi*WI (1 unit; New England Biolabs GmbH, Frankfurt/Main, Germany) in NEBuffer 3.1 (1×) for 1 h at +55°C followed by enzyme heat inactivation (20 min, +65°C). After agarose gel electrophoresis, ganglidiomab VL (407 bp) was purified from agarose gel and spectrophotometrically quantified as described above.

#### Generation of chimeric human/mouse ganglidiximab heavy and light chain

**Fig 1:**
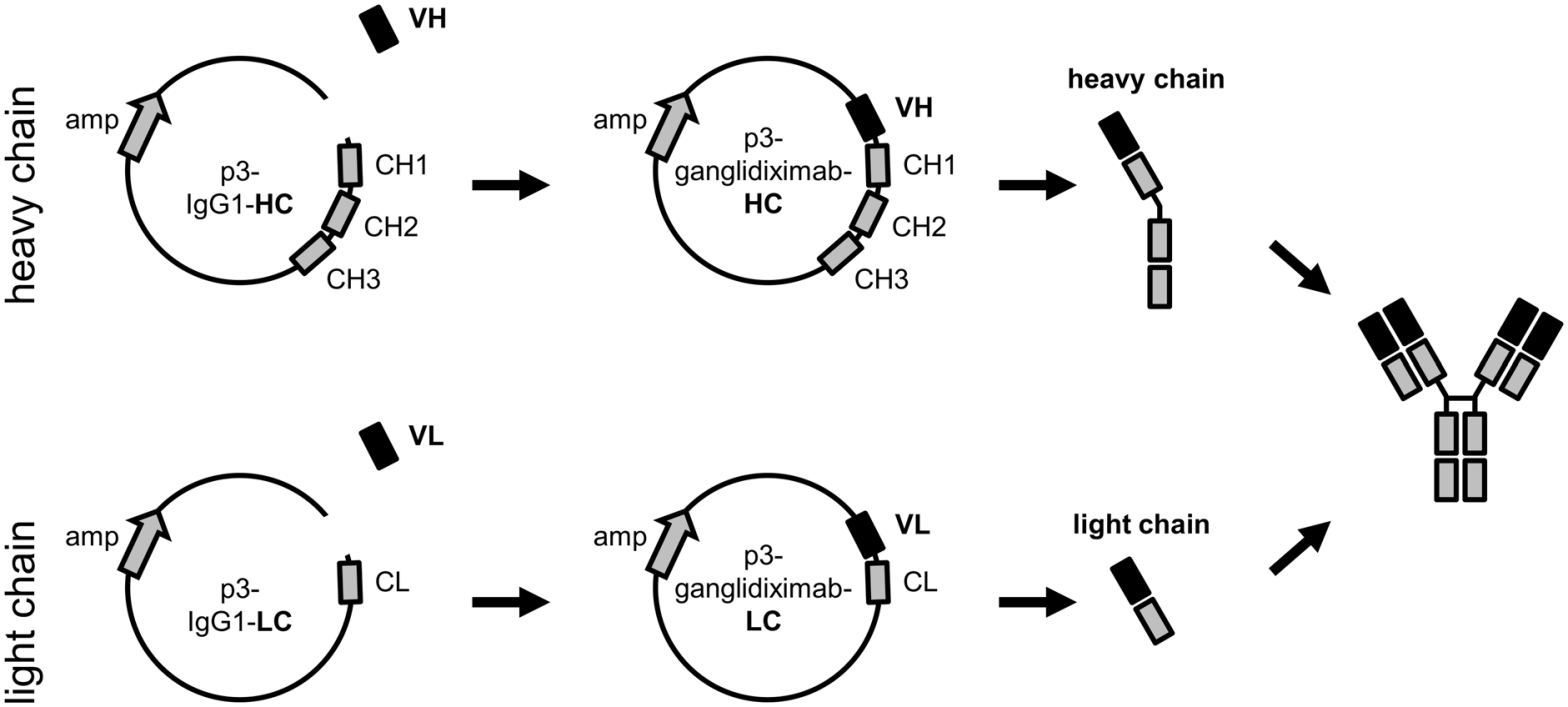
Schematic overview of generation of human/mouse chimeric anti-Id Ab ganglidiximab. The human/mouse chimeric anti-Id Ab ganglidiximab is composed of GD_2_ mimicking variable regions (VH, VL) of murine anti-Id ganglidiomab and human IgG1 constant regions. Coding sequences of VH and VL were synthesized and inserted into mammalian expression plasmids containing DNA sequences for human IgG1 heavy (p3-IgG1-HC) and light chain (p3-IgG1-LC), respectively. For ganglidiximab production, CHO cells were stably co-transfected with the two generated expression plasmids (p3-ganglidiximab-HC and p3-ganglidiximab-LC).

For ganglidiximab generation, purified ganglidiomab VH and -VL were cloned in frame into respective mammalian expression plasmids with coding sequences for human IgG1 heavy (p3-IgG1-HC) and light chain (p3-IgG1-LC), respectively (evitria AG, Zurich-Schlieren, Switzerland) (Fig 1).

First, plasmid DNA was linearized using respective restriction enzymes as described above allowing integration of respective murine variable region into the multiple cloning site (MCS). For this, linearized plasmid DNA (50 ng) and ganglidiomab variable region DNA fragment (50 ng) were incubated with Rapid Ligation Buffer (1×; Promega GmbH, Mannheim, Germany) and T4 DNA Ligase (1 unit; Promega GmbH, Mannheim, Germany) overnight at +4°C. Subsequently, 10 μl of the ligation product were used to transform NEB 10-beta chemically competent *E*. *coli* (New England Biolabs GmbH, Frankfurt/Main, Germany) according to the manufacturer's guidelines followed by overnight culturing at +37°C on LB agar plates (Roth, Karlsruhe, Germany) containing ampicillin (50 μg/ml). Resistant *E*. *coli* colonies were transferred into 5 ml LB medium (Roth, Karlsruhe, Germany) supplemented with 50 μg/ml ampicillin and cultured overnight at +37°C. Plasmids of successful transformed cells were then isolated from suspension followed by determination of DNA concentration as described above. After sequencing, sufficient amounts of plasmid DNA of correct sequence were purified as described above.

### Establishment of a cell line producing chimeric human/mouse ganglidiximab

#### Co-transfection of CHO cells with p3-ganglidiximab-HC/p3-ganglidiximab-LC

To allow permenant Ab production, a new cell line stably producing chimeric ganglidiximab was established. For this purpose, we employed CHO cells, a mammalian cell line most commonly used for industrial recombinant protein production [9]. Co-transfection of CHO cells was performed using the newly generated plasmids encoding for ganglidiximab heavy (p3-ganglidiximab-HC) or light chain (p3-ganglidiximab-LC). One day prior to co-transfection, 3× 10^5^ CHO cells were seeded in 2 ml/well of Opti-MEM^®^ I Reduced Serum Medium (Life Technologies, Darmstadt, Germany) supplemented with 3% (v/v) FCS, 10 U/ml penicillin and 0.01 mg/ml streptomycin in each well (6-well plate; Sarstedt, Nümbrecht, Germany). 2 μg of each plasmid were mixed with Opti-MEM^®^ I Reduced Serum Medium, Lipofectamine^®^ 3000 transfection reagent and P3000^™^ reagent (Life Technologies, Darmstadt, Germany) according to the manufacturer's guidelines and incubated for 5 min at RT. The entire transfection mixture was added to CHO cells followed by incubation at +37°C, 5% CO_2_ for 24 h. CHO cells co-transfected with empty plasmid controls or incubated with transfection reagent without DNA plasmids as well as non-transfected cells were utilized as negative controls. To confirm ganglidiximab production in co-transfected CHO cells, supernatants were collected 24 h after co-transfection and analyzed for ganglidiximab production using standard ELISA. First, 96-well plates (Sarstedt, Nümbrecht, Germany) were coated with 250 ng/well of murine anti-GD_2_ Ab 14G2a (100 μl per well, 0.1 M carbonate/hydrogen carbonate buffer, pH 9.6, 1 h, +37°C). After three wash steps (200 μl per well; 0.05% (v/v) Tween-20 in phosphate buffered saline (PBS, pH 7.4; PAA, Pasching, Austria)), wells were blocked with 1% (w/v) bovine serum albumin (BSA; Sigma Aldrich, Steinheim, Germany) in PBS (pH 7.4) (200 μl per well; 1 h, +37°C) and washed three times (200 μl per well; 0.05% (v/v) Tween-20 in PBS, pH 7.4). Then, samples were added and incubated for 1 h at +37°C (100 μl per well). Supernatants of CHO cells co-transfected with empty plasmid controls or incubated with transfection reagent without DNA plasmids as well as non-transfected cells served as negative controls. After three wash steps (200 μl per well; 0.05% (v/v) Tween-20 in PBS, pH 7.4) ganglidiximab binding to 14G2a was detected by horseradish peroxidase-(HRP)-conjugated goat anti-human IgG (Fc-specific) mAb as a secondary Ab (100 μl per well, 1:10,000; 1 h, +37°C) (# A0170-1ML, Sigma Aldrich, Steinheim, Germany). Plates were washed three times (200 μl per well; 0.05% (v/v) Tween-20 in PBS, pH 7.4) and 75 μl of detection reagent (R&D Systems Inc, Minneapolis, MN, USA) were added according to the manufacturer's guidelines. After 5 min, 50 μl of 2 N H_2_SO_4_ were added to stop the reaction. Absorption was determined in a Synergy HT multimode microplate reader (BioTek Germany, Bad Friedrichshall, Germany) at 450 nm.

#### Selection of a cell line stably producing ganglidiximab

To establish a cell line for permanent Ab production, CHO cells were harvested 24 h after co-transfection with p3-ganglidiximab-HC/p3-ganglidiximab-LC and limited dilutions were performed (1 cell/well in 200 μl of DMEM supplemented with stable glutamine, 4.5 g/l glucose, 10% (v/v) FCS and 1× P/S) followed by culturing for 14 days. Next, supernatants were collected and analyzed for ganglidiximab production using ELISA as described above. For each further subcloning step, clones with the highest ganglidiximab-production rate were used for limited dilutions. To establish a cell line stably producing ganglidiximab, a selected cell clone was analyzed for ganglidiximab production rate after 15 passages as well as two freeze-thaw cycles. For this, isolated RNA was used for RT-PCR analysis as described above.

### Isolation of ganglidiximab from stably transfected CHO cell line

CHO cells stably producing ganglidiximab were cultured in DMEM supplemented with stable glutamine, 4.5 g/l glucose, 10% (v/v) FCS and 1× P/S for ten days. CHO supernatant was filtered in two steps using 1.2 μm and 0.22 μm filter systems (Merck Millipore Corporation, Darmstadt, Germany). Then, Ab was concentrated with a Centrifugal Concentrator (regenerated cellulose membrane, molecular weight cut off: 30 kDa) (PALL Life Sciences, Dreieich, Germany) according to the manufacturer's guidelines resulting in a ganglidiximab concentration of 21 μg/ml determined by ELISA using 14G2a as a capture mAb (as described above). For quantification seven standard samples of known ganglidiximab concentration (25.0, 12.5, 6.25, 3.13, 1.56 and 0.78 μg/ml) were prepared in PBS (pH 7.4). Prior to incubation supernatants were diluted 1:2,000 in PBS (pH 7.4). Next, supernatants with concentrated ganglidiximab were added to the spin column containing protein G (Vivaproducts, Inc., Littleton, MA, USA) and centrifuged (300× g, 30 min, RT). After two wash steps with binding buffer (7 ml, pH 7.4, 500× g, 3 min, RT), ganglidiximab was eluted using 10 ml elution buffer (pH 2.5; Vivaproducts, Inc., Littleton, MA, USA) followed by centrifugation (500× g, 3 min, RT). Finally, neutralizing buffer (1.3 ml; pH 9.0; Vivaproducts, Inc., Littleton, MA, USA) was added to achieve a sample pH of approximately 7.5. Ganglidiximab concentration was determined using ELISA as described above.

### Binding analysis of ganglidiximab to anti-GD_2_ antibodies and NK-92tr

To evaluate anti-idiotypic characteristics, ganglidiximab binding to anti-GD_2_ Abs and NK-92tr expressing a GD_2_-specific CAR was analyzed by ELISA and flow cytometry, respectively. For binding analysis of ganglidiximab to anti-GD_2_ Abs, 96-well plates were coated with 250 ng ganglidiximab per well (100 μl, 0.1 M carbonate/hydrogen carbonate buffer, pH 9.6, 1 h, +37°C). After three wash steps (200 μl per well; 0.05% (v/v) Tween-20 in PBS, pH 7.4), wells were blocked with 1% (w/v) BSA in PBS (pH 7.4) (200 μl per well; 1 h, +37°C) and washed three times (200 μl per well; 0.05% (v/v) Tween-20 in PBS, pH 7.4). Following anti-GD_2_ Abs were diluted in PBS (pH 7.4) to final concentrations of 1.0, 0.5, 0.25, 0.13, 0.06, 0.03 and 0.015 μg/ml and incubated overnight (100 μl per well, +4°C): murine Ab 14G2a [22], chimeric Ab ch14.18/CHO containing human IgG1 constant regions and variable regions of murine 14G2a [23], ch14.18-dCH2 generated by deletion of heavy chain CH2 domain [24], humanized hu14.18K322A containing a single point mutation (K322A) in the constant region which nearly abrogates complement activation [25] as well as Ab-cytokine fusion proteins named immunocytokines consisting of a GD_2_-specific Ab linked to interleukin-2 (IL-2) (ch14.18-IL-2 and hu14.18-IL-2 [26]). After five wash steps (200 μl per well; 0.05% (v/v) Tween-20 in PBS, pH 7.4), ganglidiximab binding to anti-GD_2_ Abs was detected using biotinylated ganglidiomab as a secondary Ab diluted in 1% (w/v) BSA in PBS (pH 7.4) (100 μl per well; 1:5,000; 2 h, +37°C). Biotinylation of ganglidiomab was performed using EZ-Link^®^ Sulfo-NHS-LC-Biotin (Thermo Scientific, Erlangen, Germany) as previously described [17]. After three wash steps (200 μl per well; 0.05% (v/v) Tween-20 in PBS, pH 7.4) 100 μl of Pierce High Sensitivity NeutrAvidin-HRP (Thermo Scientific, Erlangen, Germany) diluted 1:10,000 in 1% (w/v) BSA in PBS (pH 7.4) were added and incubated for 20 min at +37°C. Then, binding of ganglidiximab to anti-GD_2_ Abs was detected as described above. ELISA signals (optical density; OD) determined with 1 μg/ml ch14.18/CHO were defined as 100% binding. Rituximab and IgG2a served as negative controls. Binding of anti-GD_2_ Abs to ganglidiximab were calculated according to the formula: binding in % = (OD 450 nm × 100%) / OD 450 nm ch14.18/CHO (1 μg/ml). For flow cytometric analysis of ganglidiximab binding to NK-92tr, 1× 10^6^ cells were harvested and washed with fluorescence-activated cell sorting (FACS) buffer (PBS (pH 7.4) supplemented with 1% (w/v) BSA, 0.1% (w/v) EDTA and 0.1% (w/v) NaN_3_; Roth, Karlsruhe, Germany) (centrifugation: 300× g, 5 min, RT). Then, cells were incubated with ganglidiximab (1 μg) for 30 min on ice (dark). Ganglidiomab served as a positive control and murine IgG1 and rituximab as isotype controls. After a further wash step, cells were incubated with 1 μg biotinylated ch14.18/CHO and washed again. PE-labeled streptavidin (BioLegend, San Diego, CA, USA) was used for detection (1:1,300; diluted in FACS buffer; 20 min, on ice, dark). Exclusion of dead cells was based on staining with DAPI (4 μl, 0.1 μg/ml, 5 min) prior to analysis. Sample acquisition was performed with a FACSCanto II flow cytometer and FACSDiva software (BD Biosciences, Heidelberg, Germany). FlowJo (Treestar, Ashland, OR, USA) was used for data analysis.

### Binding affinity of ganglidiximab to anti-GD_2_ antibodies

To further confirm anti-idiotypic characteristics, binding affinity of chimeric ganglidiximab to anti-GD_2_ Abs (analyte) was determined by surface plasmon resonance analysis using a four-channel Biacore T200 system (Biacore, Uppsala, Schweden). Additionally, we reanalyzed binding affinity of murine anti-Id Ab ganglidiomab [17]. For coupling, individual channels of a CM5 sensor chip were activated using the amine Coupling Kit (Biacore, Uppsala, Sweden). Briefly, equal volumes of 0.05 M N-hydroxysuccinimide and 0.2 M 1-ethyl-3-(3-dimethylaminopropyl)-carbodiimide hydrochloride were injected at 10 μl/min for 60 s to activate the chip surface followed by injection of the respective Ab. Ganglidiximab and ganglidiomab were diluted in acetate buffer (10 mM, pH 5.5, Biacore, Uppsala, Sweden) to a final concentration of 2.5 μg/ml. Chimeric mAb rituximab and murine IgG1 isotype control (R&D Systems Inc, Minneapolis, MN, USA) served as negative controls. Immobilization target level was defined as 300 response units (RU). Free reactive ester groups on the surface were subsequently blocked using ethanolamine hydrochloride buffer (1 M, pH 8.5, 10 μl/min for 60 s). Serial dilutions of each analyte (14G2a, ch14.18/CHO, hu14.18K322A, ch14.18-IL-2 and hu14.18-IL-2) was prepared in PBS (pH 7.4, 10 mM phosphate buffer, 2.7 mM KCl, 137 mM NaCl; Biacore, Uppsala, Sweden) (final concentrations: 128.0, 32.0, 8.0, 2.0 and 0.5 μg/ml). Between injections the sensor surface was regenerated for 30 s with 10 mM Glycine/HCL (pH 3, 30 μl/min). Binding affinity was analyzed using the “Single-Cycle-Kinetics” method. The dissociation constant (K_D_) was calculated for each Ab with BiaEvaluation software (Biacore, Uppsala, Sweden) using a steady-state fit model. Affinity measurements of both anti-Id Abs were performed in triplicates.

### Ganglidiximab-dependent competitive inhibition of anti-GD_2_ antibody binding to GD_2_

To confirm anti-Id characteristics of ganglidiximab, the previously described competitive ELISA was used to inhibit binding of anti-GD_2_ Abs (14G2a, ch14.18/CHO, ch14.18-dCH2, hu14.18K322A, ch14.18-IL-2 and hu14.18-IL-2) to the nominal antigen GD_2_ [17]. Briefly, 96-well plates were coated with 50 ng GD_2_ (Sigma Aldrich, Steinheim, Germany) per well (50 μl; 99.9% methanol (Roth, Karlsruhe, Germany); 1 h, +50°C) as a capture antigen. After methanol evaporation wells were blocked with 1% (w/v) BSA in PBS (pH 7.4) (100 μl per well; 1 h, +37°C) and washed three times (200 μl per well; 0.1% (w/v) BSA in PBS, pH 7.4). Ganglidiximab was diluted in PBS (pH 7.4) to a final concentration of 1.68 μg/ml followed by serial 1:2 dilution yielding 0.840 μg/ml (1:2), 0.420 μg/ml (1:4), 0.210 μg/ml (1:8), 0.105 μg/ml (1:16), 0.053 μg/ml (1:32), 0.026 μg/ml (1:64) and 0.013 μg/ml (1:128). Then, anti-GD_2_ Abs were applied to each dilution of ganglidiximab at a final concentration of 0.330 μg/ml and mixed. 200 μl of each dilution was added per well and incubated (2 h, +37°C). After five wash steps (200 μl per well; 0.1% (w/v) BSA in PBS, pH 7.4) anti-GD_2_ antibodies bound to GD_2_ were analyzed using biotinylated ganglidiomab as a secondary Ab diluted in 1% (w/v) BSA in PBS (pH 7.4) (100 μl per well; 1:5,000; 2 h, +37°C). After plates were washed three times (200 μl per well; 0.1% (w/v) BSA in PBS, pH 7.4), 75 μl detection reagent (R&D Systems Inc, Minneapolis, MN, USA) were added according to the manufacturer's guidelines. After 5 min, 50 μl of 2 N H_2_SO_4_ were added to stop the reaction. Absorption was determined in a Synergy HT multimode microplate reader (BioTek Germany, Bad Friedrichshall, Germany) at 450 nm. ELISA signals (OD) determined using anti-GD_2_ Abs (0.330 μg/ml) without ganglidiximab were defined as 100% binding, that is, 0% inhibition OD. Rituximab served as a negative control. Percent of binding inhibition was then calculated according to the formula: inhibition of binding % = 100%—[experimental OD / (0% inhibition OD × 100%)].

### Competitive inhibition of GD_2_-specific cytotoxicity against NB by ganglidximab

To confirm anti-idiotypic characteristics in a functional assay, GD_2_-specific NB cell lysis mediated by anti-GD_2_ Abs or NK-92tr cells expressing a GD_2_-specific CAR was evaluated using ganglidiximab as a GD_2_ surrogate [17, 27]. The cytotoxicity assay was based on the release of an acetomethoxy (AM) derivate of calcein which is a membrane-permeable live-cell labeling fluorescent dye. For NB target cell labeling, 0.6×10^6^ LA-N-1 cells were harvested and washed twice in PBS (pH 7.4) (centrifugation: 300× g, 5 min, RT). Next, the cell pellet was resuspended in heat-inactivated 12.5% (v/v) FCS in RPMI and incubated with 10 mM calcein-AM (Sigma Aldrich, Steinheim, Germany) for 30 min at +37°C shaking at 100 rpm under CO_2_-free atmosphere (dark). After two wash steps, supernatants were collected for background calculation. For analysis of Ab-mediated NB cell lysis, target cells were then resuspended in RPMI (complement-dependent cytotoxicity; CDC) or RPMI supplemented with heat-inactivated 12.5% (v/v) FCS (antibody-dependent cellular cytotoxicity; ADCC). For ADCC, 5×10^3^ LA-N-1 cells labeled with calcein-AM were incubated with ch14.18/CHO (1 μg/ml) for 30 min at +37°C in CO_2_-free atmosphere (dark). Then, effector cells of a healthy donor were incubated using an effector-to-target cell ratio (E:T) 40:1 as previously described [28] for further 4 h at +37°C in CO_2_-free atmosphere (dark). For CDC, 5×10^3^ calcein-AM-labeled LA-N-1 cells were incubated with ch14.18/CHO (1 μg/ml) and 50 μl serum of a healthy donor without effector cells for 4 h at +37°C in CO_2_-free atmosphere (dark). For evaluation of GD_2_-specific NB cell lysis mediated by NK-92tr cells, 5×10^3^ LA-N-1 cells labeled with calcein-AM as described above were incubated with NK-92tr cells (E:T 6:1) for 4 h at +37°C in CO_2_-free atmosphere (dark). Respective cell culture medium was added to achieve a final volume of 200 μl per well. To analyze ganglidiximab-dependent inhibition of GD_2_-specific target cell lysis, samples was pre-incubated with excess of ganglidiximab (5 μg/ml) for 20 min at RT. After incubation, supernatants (50 μl) of each well were transferred into a black 96-well plate (PAA, Pasching, Austria) for determination of fluorescence at 495 nm excitation and 515 nm emission wavelengths using a Synergy HT multimode microplate reader (BioTek Germany, Bad Friedrichshall, Germany). Analyses of spontaneous release (target cells only) and maximum release were further included [17]. Experiments were analyzed using six replicate wells. Cytotoxicity was calculated according to the formula: lysis % = (experimental release—spontaneous release) / (maximum release—spontaneous release) × 100%. Finally, inhibition of GD_2_-specific NB cell lysis was calculated as follows: inhibition of lysis % = 100%—[(lysis % × 100%) / (lysis % w/o anti-Id Ab)].

### Analysis of GD_2_-specific humoral immune response after vaccination with anti-idiotype ganglidiximab

Mice (n = 8) were immunized intraperitoneally (i.p.) by injection of 100 μg ganglidiximab in combination with 400 μg of adjuvant aluminum hydroxide (Invivogen, Toulouse, France). Aluminum hydroxide is a clinically approved highly effective adjuvant. It forms a long-lasting depot maintaining local antigen concentrations and promotes antigen uptake by antigen-presenting cells. Furthermore, aluminum hydroxide mainly induces a polarized T_H_2 cell (T helper cell subtype 2) response to nearly all protein antigens [29]. Control groups received Al(OH)_3_ or 0.9% NaCl. Immunization was repeated six times at 2-week intervals and serum samples were taken before and after the last immunization step.

Next, serum of immunized mice was analyzed for Abs directed against the GD_2_-mimicking paratopes of ganglidiximab based on our optimized and recently reported ELISA method [17]. In this case, 96-well plates were coated with ganglidiximab. For quantification, seven standard samples containing known concentration of murine anti-GD_2_ Ab 14G2a were prepared. Binding was detected using HRP-conjugated goat anti-mouse IgG (Fc-specific) (# A0168-1ML, Sigma Aldrich, Steinheim, Germany) as secondary mAb. To avoid quantification of Abs directed against IgG1 human constant regions, data were normalized to signals obtained using chimeric isotype rituximab as a capture Ab.

### Statistics

After testing for normality and equal variance across the experimental groups, differences were assessed with One-way ANOVA test followed by an appropriate post hoc comparison. A *P* level of < 0.05 was considered significant. All data are given as means ± SD or means ± SEM. Analysis was performed using the software SigmaStat (Jandel, San Rafael, CA).

## Results

### Generation of chimeric anti-idiotype antibody ganglidiximab

**Fig 2:**
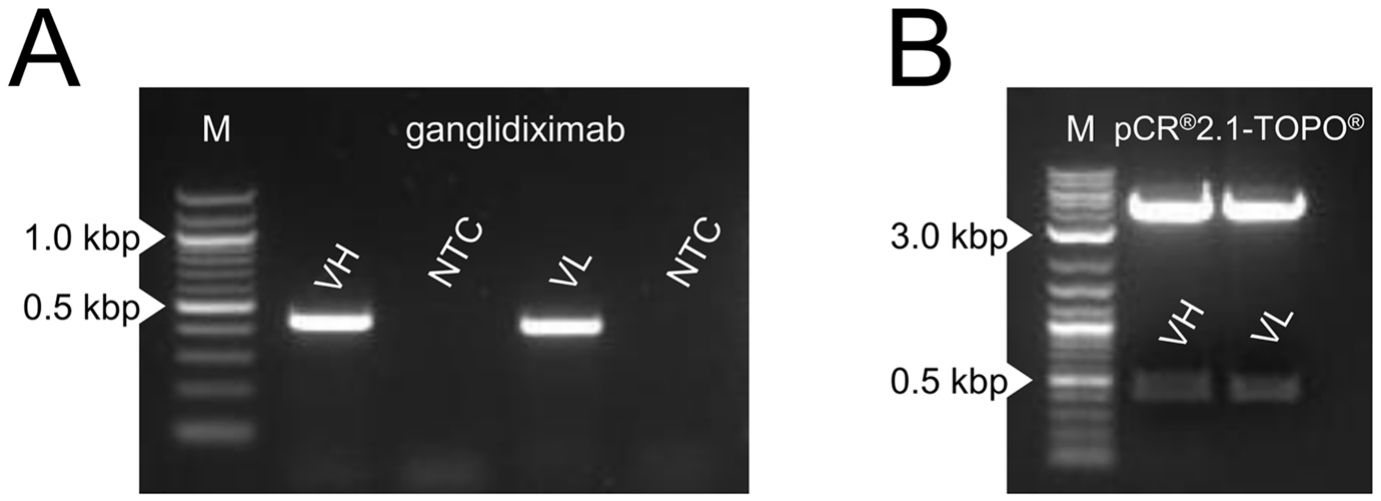
Amplification of DNA fragments encoding for GD_2_-mimicking paratopes of ganglidiximab. (**A**) Visualization of coding sequences of GD_2_-mimicking variable heavy (VH; 440 bp) and light chain (VL; 420 bp) amplified by RT-PCR. RNA was isolated from hybridoma cells producing murine anti-Id ganglidiomab. PCR products were analyzed by agarose gel electrophoresis. Representative image is shown. NTC—no-template-control. M—Marker (100-bp). (**B**) PCR products were cloned into pCR^®^2.1-TOPO^®^ plasmids and analyzed by restriction enzyme digest to excise DNA sequences encoding for VH and VL (product sizes 427 bp and 407 bp, respectively). Resulting DNA fragments were analyzed by agarose gel electrophoresis. Representative image is shown. M—Marker (2-log, 0.1–10.0 kbp).

The newly generated chimeric anti-Id Ab ganglidiximab is composed of murine variable regions (VH, VL) derived from previously reported anti-Id ganglidiomab [17] and human instead of mouse IgG1 constant regions. First, coding sequences of murine GD_2_-mimicking VH and VL were amplified by RT-PCR using RNA isolated from hybridoma cells producing murine anti-Id ganglidiomab (Fig 2A). PCR products were cloned into pCR^®^2.1-TOPO^®^ plasmids for sequencing. The sequences of frameworks and complementary-determining regions of VH and VL were found to be identical to the previously reported sequences of ganglidiomab [17]. Next, VH and VL were excised from pCR^®^2.1-TOPO^®^ plasmids by restriction enzyme digest revealing DNA fragments of expected size of 427 bp for VH and 407 bp for VL (Fig 2B). Further, VH and VL were successfully ligated in frame into respective mammalian expression plasmids with coding sequences for human IgG1 heavy and light chain, respectively. After transformation in *E*. *coli*, plasmids from 14 selected clones were isolated and subjected to further experimental steps. Sequence analysis revealed identical VH and VL sequences compared to the respective variable regions of ganglidiomab [17]. Finally, the two generated plasmids encoding for ganglidiximab heavy (p3-ganglidiximab-HC) and light chain (p3-ganglidiximab-LC) were used for further Ab production.

### Establishment of a cell line stably producing ganglidiximab

**Fig 3:**
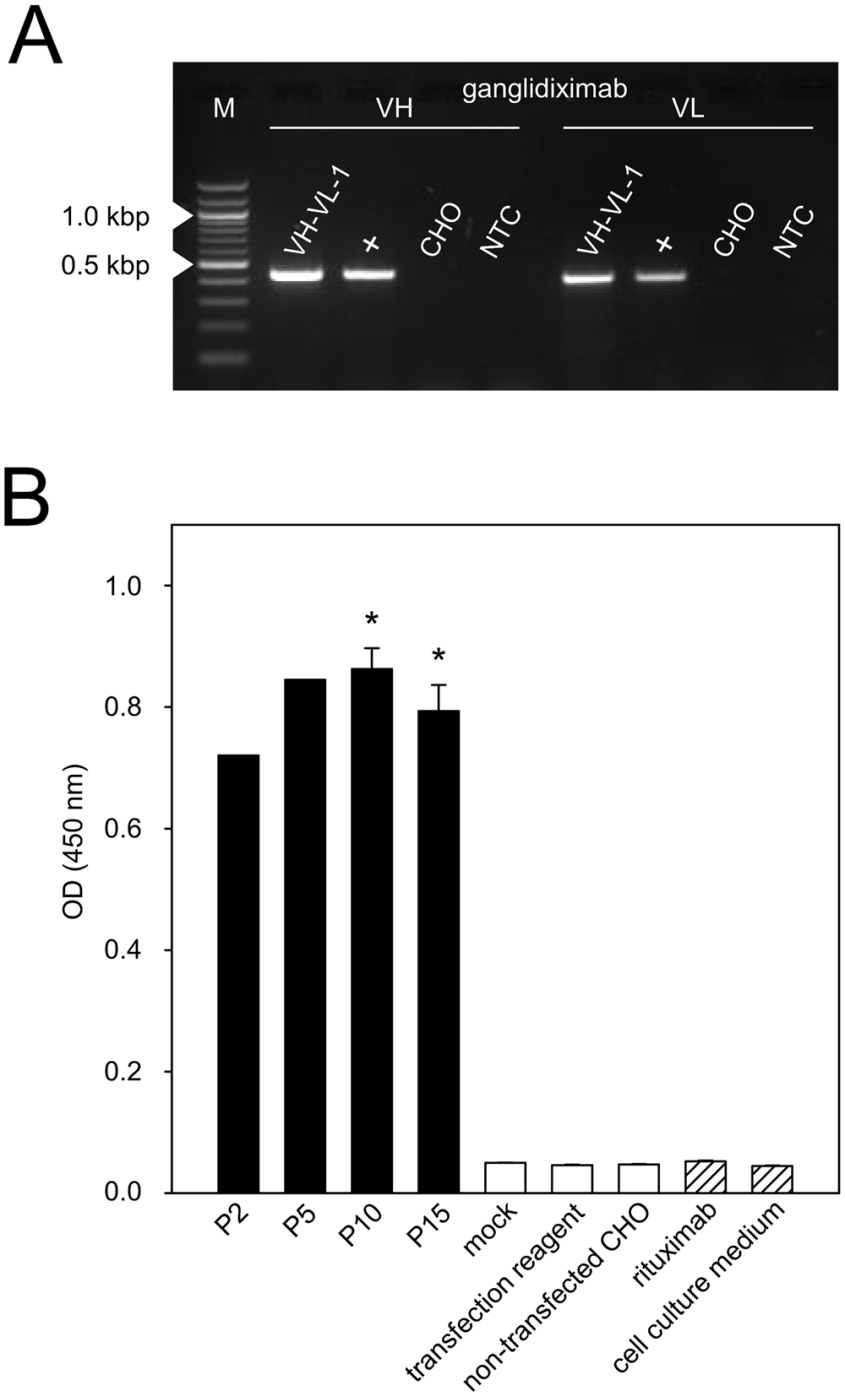
Establishment of a cell line stably producing ganglidiximab. CHO cells were stably co-transfected with two generated expression plasmids (p3-ganglidiximab-HC/ p3-ganglidiximab-LC) and a cell clone “VH-VL-1” stably producing high levels of ganglidiximab was selected for further Ab production. Permanent ganglidiximab expression was confirmed after 15 passages and two freeze-thaw cycles by RT-PCR (A) and standard ELISA (B). (**A**) To amplify coding sequences of ganglidiximab VH (440 bp) and -VL (420 bp), RNA of “VH-VL-1” was used for RT-PCR followed by agarose gel electrophoresis. RNA of ganglidiomab-producing hybridoma cells served as a positive control (+) and RNA of non-transfected CHO cells as a negative control. One representative image is shown. NTC—no-template-control. M—Marker (100-bp). (**B**) Ganglidiximab production by “VH-VL-1” was analyzed in supernatants collected during 15 passages and after two freeze-thaw cycles (P2, 5, 10 and 15). Supernatants of non-transfected CHO cells or cells incubated with control plasmids (mock) or transfection reagent only were utilized as negative controls (white columns). Human/mouse chimeric mAb rituximab and cell culture medium were included as additional negative controls (white-striped columns). Data are shown as mean values ± SEM of three independent experiments performed at least in triplicates. One-way ANOVA on ranks followed by appropriate post hoc comparison test; *P 0.05 *vs*. negative controls.

To establish a cell line allowing permanent ganglidiximab production, CHO cells were stably co-transfected with the two generated expression plasmids (p3-ganglidiximab-HC, p3-ganglidiximab-LC). After five rounds of subcloning one cell clone (“VH-VL-1”) was found to produce high levels of ganglidiximab. Then, analysis of permanent Ab production was performed. For this purpose, the selected cell clone was subjected to 15 passages as well as two freeze-thaw cycles, which are procedures known to provoke shedding of plasmids not stably integrated into the host genome. RT-PCR analysis performed after 15 passages and the second freeze-thaw cycle revealed stable mRNA expression of both murine heavy and light chain variable region in “VH-VL-1” clone (Fig 3A). ELISA analysis of supernatants collected in early (P2 and 5) and late passages (P10 and15) revealed similar levels of Ab concentration (*P < 0.05 *vs*. negative controls; Fig 3B) indicating a stable Ab production over time. Importantly, two freeze-thaw cycles performed between passages 10 and 15 did not affect ganglidiximab production rate confirming stable integration of plasmid DNA into the host genome of selected cell clone. Finally, ganglidiximab production was quantified in supernatants collected after 15 cell clone passages and two freeze-thaw cycles showing about 3 μg/ml ganglidiximab prior to and 21 μg/ml after the Ab concentration procedure. The selected cell clone named “VH-VL-1” was used for Ab purification and further analysis.

### Analysis of anti-idiotypic characteristics of ganglidiximab

#### Binding of anti-GD_2_ antibodies to ganglidiximab

**Fig 4:**
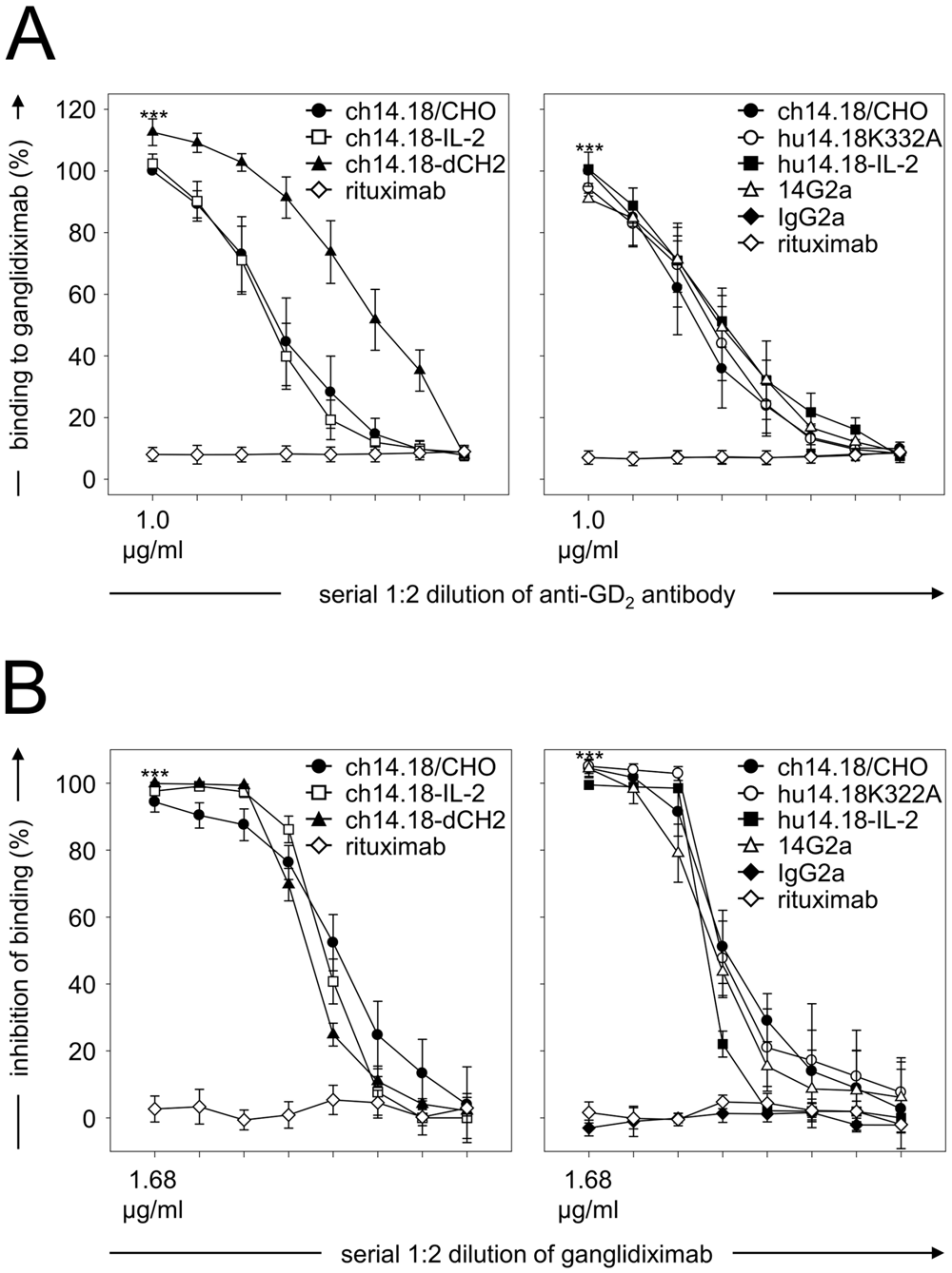
Binding of anti-GD_2_ antibodies to ganglidiximab and competitive inhibition of binding to nominal antigen GD_2_. (**A**) Concentration-dependent binding of ganglidiximab to anti-GD_2_ Abs of the 14.18 family was analyzed by ELISA. Chimeric mAb rituximab and murine IgG2a were utilized as isotype controls. Data are expressed relative to binding of 1 μg/ml of ch14.18/CHO to ganglidiximab (100%) and are presented as mean values ± SEM of four independent experiments performed in duplicates. One-way ANOVA followed by appropriate post hoc comparison test; ***P 0.001 *vs*. rituximab. (**B**) Inhibition of anti-GD_2_ Ab binding to GD_2_ by ganglidiximab was analyzed using competitive ELISA. Chimeric mAb rituximab and murine IgG2a served as isotype controls. Data are expressed as percentage of binding inhibition and presented as mean values ± SEM of four independent experiments performed in duplicates. One-way ANOVA followed by appropriate post hoc comparison test; ***P 0.001 *vs*. rituximab.

**Fig 5:**
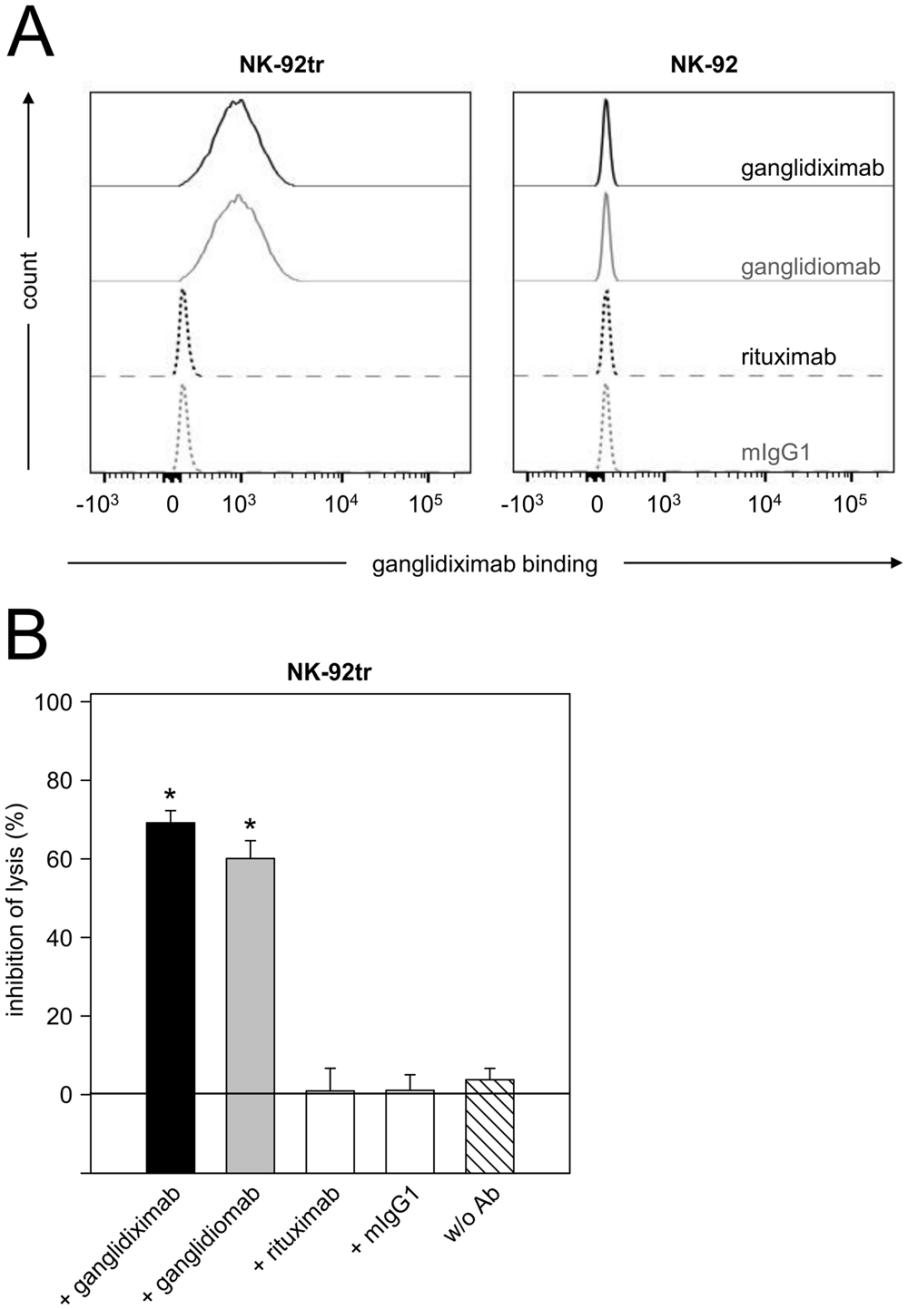
Binding of ganglidiximab to NK-92tr and ganglidiximab-dependent inhibition of NK-92tr-mediated GD_2_-specific cytotoxicity against NB. (**A**) Binding of ganglidiximab to NK-92tr expressing a GD_2_-specific chimeric antigen receptor was analyzed by flow cytometry. Cells were stained with chimeric ganglidiximab (black solid line), murine anti-Id ganglidiomab (positive control; grey solid line), chimeric rituximab (isotype control; black dashed line) or murine IgG1 (isotype control; grey dashed line) followed by incubation with biotinylated ch14.18/CHO and PE-labeled streptavidin. Staining of the parental NK-92 cell line lacking GD_2_-specific CAR expression was further included as negative control. Results from one representative experiment are shown. (**B**) Inhibition of GD_2_-specific NK-92tr-mediated NB cell lysis (w/o Ab; white-striped column) was analyzed after pre-incubation with excess of ganglidiximab (black column) using a calcein-AM-based cytotoxicity assay. Murine anti-Id ganglidiomab served as a positive control (grey column). Rituximab (white column) and murine IgG1 (white column) were utilized as negative controls. Results are expressed as percentage of lysis inhibition (mean values ± SEM) of two independent experiments performed using six replicates. One-way ANOVA on ranks followed by appropriate post hoc comparison test; *P 0.05 *vs*. w/o Ab.

Binding of anti-GD_2_ Abs of the 14.18 family to ganglidiximab was analyzed relative to the negative control rituximab using our previously described ELISA protocol [17]. Similar concentration-dependent binding of anti-GD_2_ Abs 14G2a, ch14.18/CHO, ch14.18-delta-CH2, hu14.18K322A as well as immunocytokines ch14.18-IL-2 and hu14.18-IL-2 to ganglidiximab (***P < 0.001 *vs*. rituximab; Fig 4A) was observed, indicating anti-idiotypic properties of the newly generated Ab. These results could be confirmed by flow cytometric analysis using a NK-92tr cell line expressing a GD_2_-specific chimeric antigen receptor which consists of a ch14.18-derived single chain variable region and a CD3-zeta chain [21] (Fig 5A). Similar binding of ganglidiximab and murine parental mAb ganglidiomab to NK-92tr was observed. As expected, neither ganglidiximab nor ganglidiomab bound to the parental NK-92 control cell line lacking CAR expression (Fig 5A).

#### Binding affinity of ganglidiximab to anti-GD_2_ Abs

Binding affinity of ganglidiximab to anti-GD_2_ Abs of 14.18 family (14G2a, ch14.18/CHO, ch14.18-IL-2, hu14.18K322A and hu14.18-IL-2) was further investigated by surface plasmon resonance analysis using a steady-state fit model for calculation of dissociation constant (K_D_). K_D_ of ganglidiximab from anti-GD_2_ Abs were in the range of 21.12 ± 3.18 to 166.30 ± 16.82 nM and found to be similar to binding affinities of ganglidiomab to anti-GD_2_ Abs ranging from 19.87 ± 3.44 to 134.92 ± 32.66 nM (Table 1).

**Table 1:** Dissociation constants (K_D_) of ganglidiximab and ganglidiomab from anti-GD_2_ antibodies of the 14.18 family.

Ligand	ganglidiximab KD (nM)	ganglidiomab KD (nM)
14G2a	95.43 ± 5.27	99.43 ± 6.52
ch14.18/CHO	38.40 ± 2.74	39.04 ± 3.14
ch14.18-IL-2	166.30 ± 16.82	134.92 ± 32.66
hu14.18K322A	32.58 ± 4.36	33.36 ± 4.16
hu14.18-IL-2	21.12 ± 3.18	19.87 ± 3.44

Dissociation constants (K_D_) of ligands 14G2a, ch14.18/CHO, ch14.18-IL-2, hu14.18K322A and hu14.18-IL-2 from ganglidiximab and ganglidiomab were determined in a Biacore T200 system by surface plasmon resonance method using a steady-state fit model. Values are presented as mean values ± SEM of three independent experiments.

#### Ganglidiximab-dependent inhibition of binding of anti-GD_2_ antibodies to GD_2_

Binding of anti-GD_2_ Abs (14G2a, ch14.18/CHO, ch14.18-IL-2, hu14.18K322A and hu14.18-IL-2) to the nominal antigen GD_2_ was competitively inhibited by ganglidiximab in a concentration-dependent manner (***P < 0.001 *vs*. rituximab; Fig 4B), again clearly indicating anti-idiotypic characteristics of ganglidiximab.

#### Ganglidiximab-dependent inhibition of anti-GD_2_ Ab-mediated GD_2_-specific neuroblastoma cell lysis

**Fig 6:**
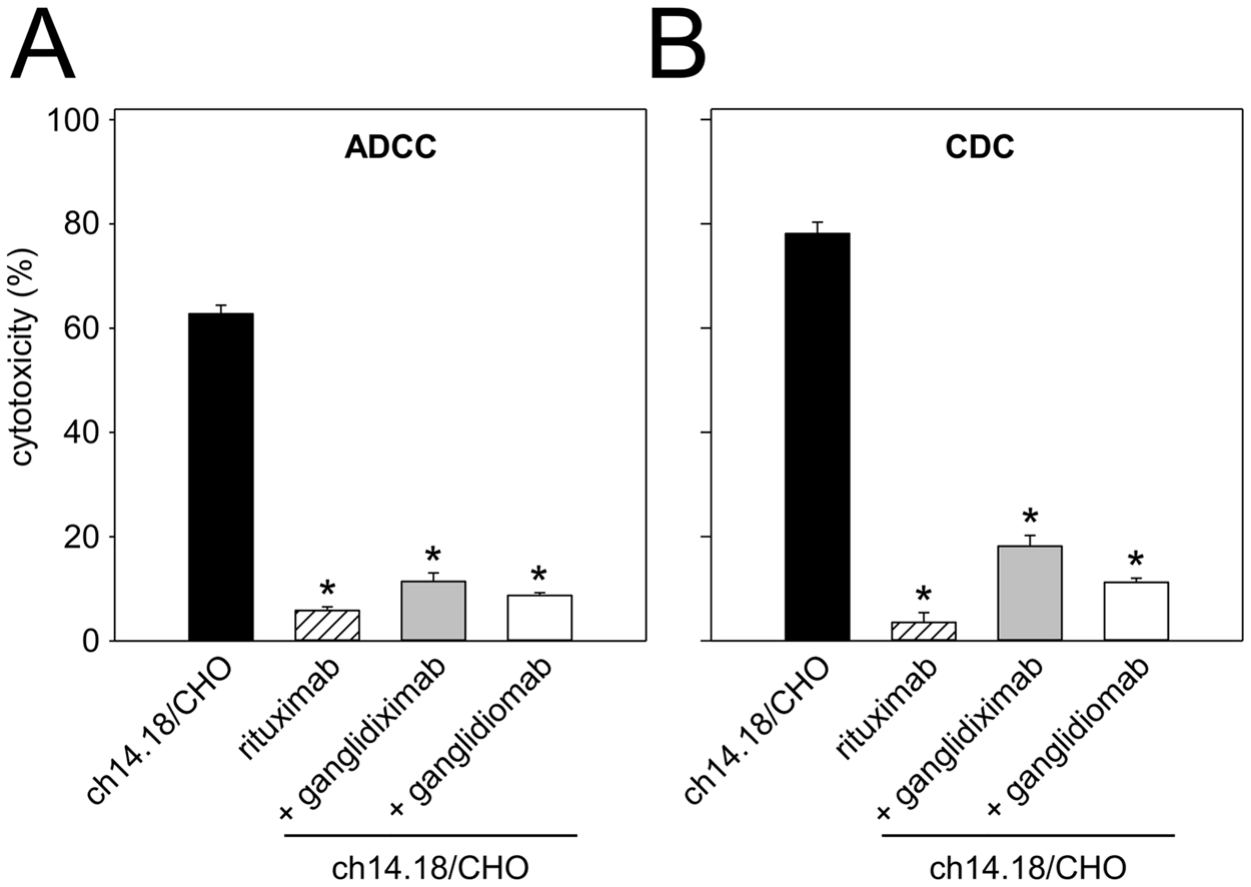
Ganglidiximab-dependent inhibition of GD_2_-specific ch14.18/CHO-mediated ADCC and CDC. GD_2_-specific ch14.18/CHO-mediated ADCC (**A**) and CDC (**B**). Ch14.18 induced ADCC and CDC of NB cells LA-N-1 (black column) was compared to rituximab used as negative control (white-striped column). Pre-incubation of ch14.18 with excess of ganglidiximab (grey column) or ganglidiomab (white column) resulted in inhibition of both ADCC and CDC. Results are expressed as percentage of cytotoxicity (mean values ± SEM) of three independent experiments performed at least in triplicates. One-way ANOVA on ranks followed by appropriate post hoc comparison test; *P 0.05 *vs*. ch14.18/CHO.

GD_2_-mimicking characteristics of ganglidiximab were further evaluated with a functional calcein-AM-based cytotoxicity assay as previously described [17, 27, 28]. Healthy donor leukocytes or NK-92tr cells and serum of a healthy donor were used for ADCC and CDC, respectively. Pre-incubation with excess of ganglidiximab resulted in a significant inhibition of cytotoxic activity mediated by healthy donor leukocytes (“+ ganglidiximab” 11.4 ± 1.6% *vs*. “ch14.18/CHO” 62.8 ± 1.6%; *P 0.05; Fig 6A) and NK-92tr. cells (“+ ganglidiximab” 69.2 ± 3.1% *vs*. “+ rituximab” 0.9 ± 5.8%; *P 0.05; Fig 5B) or complement proteins (“+ ganglidiximab” 18.1 ± 2.0% *vs*. “ch14.18/CHO” 78.1 ± 2.1%; *P 0.05; Fig 6B). In summary, these results confirm anti-idiotypic properties of ganglidiximab.

### Induction of a GD_2_-specific humoral immunity following vaccination with ganglidiximab

**Fig 7:**
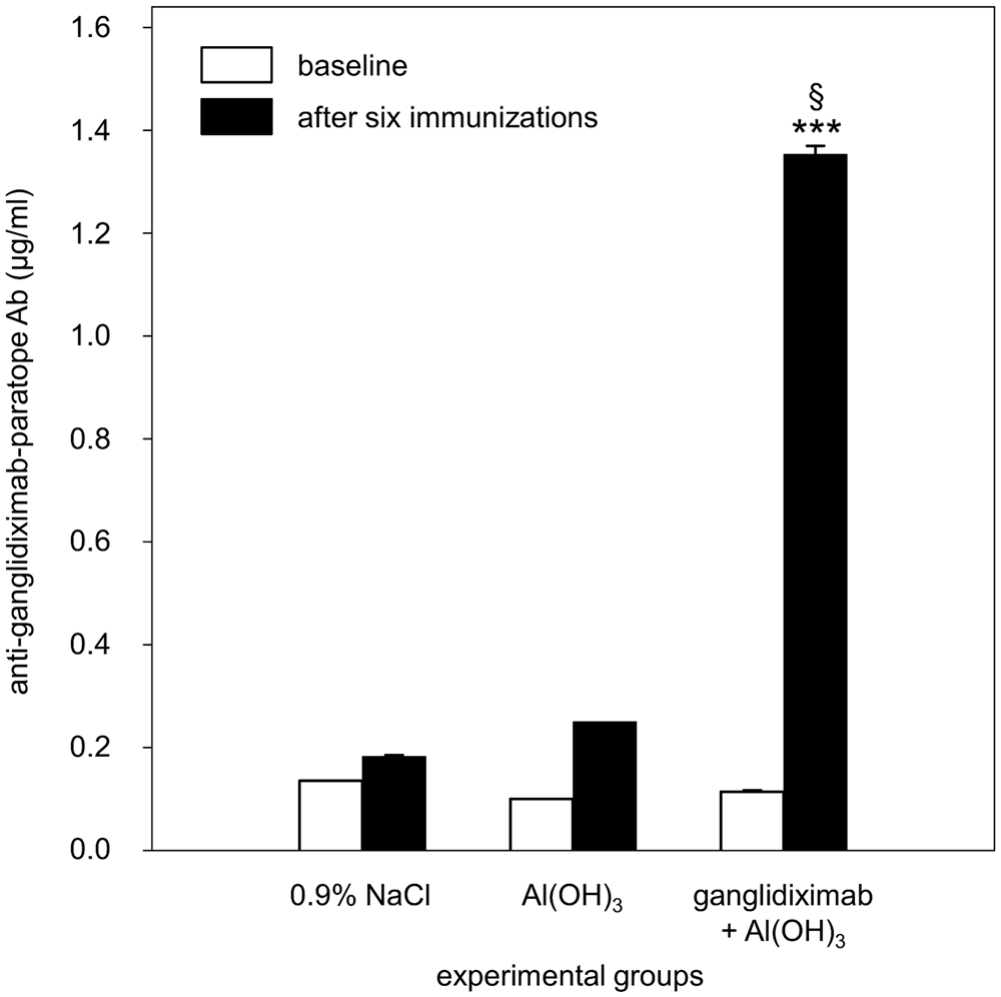
Induction of GD_2_-specific humoral immunity after vaccination with ganglidiximab. Female A/J mice (n = 8) were immunized six times with ganglidiximab combined with the adjuvant Al(OH)_3_ every two weeks. Control groups received Al(OH)_3_ or 0.9% NaCl. Serum samples were taken before (baseline; white column) and after the last immunization step (black column) and analyzed for Abs directed against GD_2_-mimicking paratopes of ganglidiximab using ELISA. Mann-Whitney Rank Sum test or One-way ANOVA followed by appropriate post hoc comparison test; ***P 0.001 *vs*. baseline (prior to the first immunization); ^§^*P* < 0.05 vs. NaCl control group after six immunizations.

Induction of a GD_2_-specific humoral immune response *in vivo* was investigated after vaccination of mice with the chimeric anti-Id Ab ganglidiximab. For this purpose, A/J mice received ganglidiximab in combination with the adjuvant Al(OH)_3_. Serum samples were obtained before (baseline) and after the last immunization and analyzed for induction of GD_2_-specific Abs by ELISA as described in “Material and methods” section. Mice immunized with the combination of ganglidiximab and Al(OH)_3_ clearly showed induction of a GD_2_-specific humoral immunity compared to control groups which received Al(OH)_3_ or 0.9% NaCl (“ganglidiximab + Al(OH)_3_” 1.35 ± 0.02 μg/ml *vs*. “Al(OH)_3_” 0.25 ± 0.0 μg/ml *vs*. “0.9% NaCl” 0.18 ± 0.0 μg/ml; *P 0.05; Fig 7). Importantly, we detected a significant increase of Abs directed against the GD_2_-mimicking paratopes compared to the baseline (Fig 7), demonstrating the efficacy of protein vaccination with ganglidiximab *in vivo*.

## Discussion

Immunotherapy targeting GD_2_ is an encouraging approach to improve survival in high-risk NB patients. Therefore, GD_2_-specific mAbs have been developed and successfully applied in several clinical trials [1;7;9;10]. Since passive immunotherapy generally does not induce immunological memory, there is a considerable interest in extending these studies into active immunotherapy. However, due to its poorly immunogenic properties, the nominal antigen GD_2_ used as a vaccine elicited only low-titer IgM immune responses with decreased anti-GD_2_ activity over time [17, 30]. Hence, the use of GD_2_ surrogate protein vaccines provides an alternative approach against GD_2_-expressing neuroectodermal tumors given that they are chemically stable, can be economically produced and do not contain oncogenic or toxic material [14]. In previous studies, GD_2_ epitopes translated into immunogenic peptides such as GD_2_ peptide mimotopes have shown promising results in experimental models [31] and are currently under development for the use in patients. Here, we report another promising alternative strategy which exploits the immune network hypothesis of Jerne [13] to mimic GD_2_ as a protein epitope by an anti-Id Ab. Acting as a TAA surrogate, anti-Id Abs were found to be associated with improved patient survival as they can induce generation of anti-anti-Id Abs recognizing the nominal TAA and may therefore facilitate host recognition of the tumor [14, 32]. Recently, successful vaccination with anti-Id Ab has been shown in treatment of melanoma, lung cancer, B cell lymphoma and leukemia [14]. For NB treatment, Yu *et al*. conducted a clinical trial of GD_2_-mimicking anti-Id Ab 1A7 and showed little toxicity of anti-Id protein vaccines and effective induction of biologically active GD_2_-specific immune responses [1]. To provide unrestricted access of a GD_2_-mimicking anti-Id Ab in Europe, we previously generated a new murine anti-Id Ab ganglidiomab and successfully used it as a protein vaccine to induce GD_2_-specific humoral immunity in mice [17]. Once applied in humans, the immune response induced against a mouse IgG1 mAb will be directed against the entire xenogeneic protein. In order to tailor humoral immunity towards GD_2_-mimicking paratopes for future clinical trials, we exchanged murine with human constant regions and generated the chimeric human/mouse anti-Id Ab ganglidiximab, consisting of human IgG1 constant regions, which are not immunogenic in humans, and murine variable regions derived from anti-Id ganglidiomab [17]. To allow permanent ganglidiximab production, two expression plasmids encoding for heavy and light chains were designed and stably co-transfected into CHO cells, the most widely used mammalian cell line for recombinant protein production [9, 33].

After Ab purification, we demonstrated GD_2_-mimicking properties of ganglidiximab which were found to be comparable to a previously reported murine anti-Id Ab ganglidiomab [17]. Similar to GD_2_, ganglidiximab specifically binds to clinically used Abs of the 14.18 family (14G2a, ch14.18/CHO, ch14.18-dCH2, hu14.18K322A as well as immunocytokines ch14.18-IL-2 and hu14.18-IL-2) (Fig 4A) as well as to a GD_2_-specific CAR expressed on genetically engineered NK-92 cells (NK-92tr) [21] (Fig 5A). Furthermore, GD_2_-mimicking properties were confirmed by ganglidiximab-dependent competitive inhibition of binding of anti-GD_2_ Abs to GD_2_. Then, medium binding affinities of ganglidiximab to anti-GD_2_ Abs of 14.18 family were determined using a surface plasmon resonance technique (between 10^−7^ and 10^−8^ M; Tab. 1) and were found to be similar to the previously generated murine anti-Id ganglidiomab [17]. To further confirm ganglidiximab function as a GD_2_ surrogate, we applied a recently reported functional cytotoxicity assay [17, 28], demonstrating ganglidiximab-dependent inhibition of a GD_2_-specific NB cell lysis mediated by effector cells (ADCC) and serum of a healthy donor (CDC; Fig 6). These data are in line with our previous results clearly showing similar anti-idiotypic properties of both anti-Id Abs [17, 28]. Finally, as it has already been shown for murine anti-Id Abs *in vivo* [16, 17], ganglidiximab was used as a GD_2_ surrogate protein vaccine to induce a humoral immunity towards the GD_2_-mimicking paratopes in mice (Fig 7). For future clinical application, we plan a combined two-phase anti-Id-based vaccination strategy starting with murine and followed by human/mouse chimeric anti-Id Abs. In humans, the murine ganglidiomab Fc portion probably serves as an immunogenic carrier [14] thereby priming a GD_2_-specific humoral response upon repeated vaccination. In the second phase, vaccination with the chimeric ganglidiximab, containing non-immunogenic human constant regions, may tailor humoral immunity towards the therapy-relevant paratopes mimicking GD_2_.

In summary, we describe the generation and characterization of a new chimeric anti-Id Ab ganglidiximab used as a protein vaccine to induce humoral immunity against NB in mice. Our results are an important baseline for further development of new anti-Id Ab-based vaccination approaches against GD_2_-expressing malignancies.
